# Effect of Statins in the Watch and Wait Phase of Chronic Lymphocytic Leukemia

**DOI:** 10.1002/cam4.70881

**Published:** 2025-04-18

**Authors:** David E. Spaner

**Affiliations:** ^1^ Biology Platform Sunnybrook Research Institute Toronto Canada; ^2^ Department of Immunology University of Toronto Toronto Canada; ^3^ Department of Hematology Odette Cancer Center Toronto Canada; ^4^ Department of Medical Biophysics University of Toronto Toronto Canada; ^5^ Dept. of Medicine University of Toronto Toronto Canada

**Keywords:** chronic lymphocytic leukemia, lipoproteins, STAT3, statins

## Abstract

**Background:**

It is an unclear how cholesterol‐lowering statin drugs affect progression of chronic lymphocytic leukemia (CLL).

**Methods:**

Clinical records of 57 CLL patients were examined to determine how initiating statins in the “watch and wait” phase of management affected disease progression.

**Results:**

After 6.4 ± 0.6 months, when average low‐density lipoprotein cholesterol levels had been lowered from 3.58 ± 0.11 mM to 2.1 ± 0.06 mM, blood levels of CLL cells and beta‐2‐microglobulin (β2M) increased significantly, accompanied by significant decreases in platelets. Following statin institution, rates of change of blood lymphocytes and β2M increased from 1.55 ± 0.39 × 10^6^ to 3.4 ± 0.68 × 10^6^ cells/mL/month (*n* = 43) and 0.035 ± 0.011 to 0.055 ± 0.007 μg/mL/month (*n* = 40), respectively. Conventional first‐line CLL treatment was ultimately required in 37 patients.

**Conclusions:**

These observations suggest that statins as single agent do not slow and may even modestly stimulate progression of CLL.

A passive “watch and wait” approach continues to be employed for asymptomatic patients diagnosed with chronic lymphocytic leukemia (CLL) [1]. Once symptoms develop, novel agents like Bruton's tyrosine kinase and BCL‐2 inhibitors improve outcomes but do not cure the disease. A drug that could prolong progression free survival (PFS) and time to first treatment during “watch and wait” might alleviate anxiety, prevent clonal evolution, and perhaps extend patient survival [2]. This possibility might be met by exploiting the anticancer properties of statins [3].

Statins prevent cardiovascular complications by lowering low‐density lipoprotein (LDL) cholesterol in the blood. By inhibiting HMG‐CoA reductase, the rate‐limiting enzyme of the mevalonate pathway, statins may also affect oncogenic pathways [3]. Lipophilic statins, such as simvastatin, can enter and affect leukemia cells, directly while hydrophilic statins like rosuvastatin act mainly in the liver to lower blood cholesterol [3]. Statins are among the most potent FDA‐approved drug classes that kill CLL cells in vitro [4]. Their well‐defined mechanism of action and safety profile suggest statins might be repurposed as effective anticancer drugs, especially for CLL [3].

Patients with CLL are reported to have unusually high rates of hypercholesterolemia [5, 6]. Prior to the modern treatment era, statin use was associated with prolongation of PFS and even improved overall survival [5, 6]. However, these findings were not observed consistently in other studies [7, 8]. One reason for these discrepancies may be that epidemiological studies are plagued by problems, such as reverse causation [9]. Moreover, they often do not account for the type of statin used (i.e., lipophilic vs. hydrophilic) or provide evidence for the biological activity of the statin, such as documenting the magnitude of reduction in LDL levels [3]. Interventional phase III trials are not available to settle the issue of whether statins can slow the progression of CLL.

To approximate potential findings of an interventional trial, a chart review was carried out of patients attending the Sunnybrook CLL clinic between 2012 and 2023. The study was approved by the Research Ethics Board of Sunnybrook Hospital (PIN 222‐2014). Fifty‐seven patients (26 female and 31 male) in the watch and wait phase of management were identified who were started on a statin (mainly simvastatin) by their oncologist, cardiologist, or family doctor after the diagnosis of CLL and where serial LDL measurements were available to assure biological activity of the drug. Patient characteristics are described in Table 1. After an average of 6.4 ± 0.6 months on statin treatment sufficient to lower LDL cholesterol from an average of 3.6 ± 0.1 to 2.1 ± 0.06 mM, white blood cells (comprised mainly of CLL cells) consistently increased while platelets decreased (Figure 1A left panel; B). β2M levels that reflect disease burden [1] also increased following institution of statins (Figure 1A left panel; B). The changes were mild but significant. Surprisingly, the rate of change of lymphocyte numbers and β2M levels increased significantly following statin therapy (Figure 1C,D). An example is provided to illustrate the temporal rise in lymphocytes and β2M levels associated with the fall in LDL levels (Figure 1A, left panel). The data set was too small to indicate if significant reversal of the changes occurred upon stopping statins. Increases in lymphocytes and β2M levels tended to be higher in patients whose CLL cells expressed unmutated *IGHV* genes (U‐CLL) compared to those with mutated genes (M‐CLL) (Figure 1B). U‐CLL is generally more aggressive than M‐CLL [1]. Of the 57 patients treated with statins, all of whom were initially managed with a “watch and wait” approach, only 20 (18 with M‐CLL and 2 with U‐CLL) did not progress to require first‐line therapy (Table 1).

**TABLE 1:** Patient characteristics.

	Pt.	Age	Sex	*IGHV*	FISH	LDL		Time	WBC		Plt		β2M		Tx	Statin
						Pre	Post		Pre	Post	Pre	Post	Pre	Post		
**Statin patients**																
	1	64	M	m	13q	4.14	2.07	2	14	16	148	127	1.7	1.7	no	sim
	2	62	F	u	13q	4.16	2.35	3	45	45.3	218	187	1.7	1.5	BKi	sim
	3	73	F	m	13q	4.4	2.93	10	20	23	163	137	3.6	3.9	no	sim
	4	69	M	u	na	3.9	2.59	7	8	10	163	154	1.7	1.8	BKi	sim
	5	69	M	m	na	3.56	2.36	11	14	16	146	144	1.5	1.5	no	sim
	6	66	M	m	13q	3.58	1.97	11	29	28	135	102	1.9	1.9	fcr	sim
	7	74	M	m	na	3.55	2.32	3	8	11	172	161	1.3	1.4	no	sim
	8	68	F	m	na	3.05	1.69	2	52	47	188	167	2	2	no	sim
	9	58	M	m	na	3.05	2.38	3	99	100	144	136	2	2.2	no	sim
	10	59	M	m	na	4.9	2.28	16	16	25	353	274	2.2	2.3	no	sim
	11	64	M	u	neg	3.1	1.73	7	60	132	244	242	2.7	4.1	BKi	sim
	12	70	M	m	13q	4.11	2.99	5	12.4	14.7	90	105	na	na	fcr	sim
	13	56	F	m	13q	4.47	2.02	10	359	328	154	124	2.6	2.5	fcr	ros
	14	70	M	u	neg	2.39	1.5	3	64	88	180	156	3.1	3	fcr	sim
	15	54	F	u	na	3.53	2.15	4	0.8	1	190	216	1.8	1.8	BKi	sim
	16	65	M	u	neg	2.89	1.72	3	60	80	86	72	3	3.1	fcr	sim
	17	63	M	u	neg	4.26	2.17	2	26	35	123	108	2.9	3	BR	sim
	18	68	M	m	11q	2.43	1.7	8	46	54	86	93	2.2	2.2	CO	sim
	19	57	M	m	13q	2.56	2.11	3	202	199	89	8	3.7	3.2	fcr	sim
	20	70	M	u	na	4.2	2.8	6	72	79	377	339	2.4	2.4	no	sim
	21	63	F	u	11q	3.16	1.46	5	16	29	211	139	5.6	6.3	fcr	ros
	22	46	M	m	neg	4.93	2.53	6	70	90	99	94	2.4	2.6	no	sim
	23	64	F	m	na	5.49	2.16	28	30	36	383	336	1.7	1.9	no	ros
	24	56	M	u	neg	4.24	2.04	8	105	154	170	120	2	2.1	fcr	sim
	25	74	F	m	neg	4.27	2.05	12	77	132	157	128	3.8	4.2	CO	sim
	26	60	F	u	neg	2.38	1.82	4	68	72	193	169	3	3.9	fcr	sim
	27	64	M	m	13q	1.28	1.99	4	104	122	133	116	3.2	3.4	CO	sim
	28	75	F	m	neg	3.37	1.63	9	71	128	97	93	5	6.5	CO	sim
	29	64	M	u	neg	3.66	1.86	4	46	67	219	179	3.3	3.9	fcr	sim
	30	59	M	m	13q	3.66	1.62	11	63	75	176	168	2.7	2.8	no	sim
	31	68	F	m	13q	2.72	1.2	7	123	137	184	217	3.3	3.4	FR	sim
	32	66	F	u	na	2.98	1.75	2	20	35	163	154	2.4	2.3	C	sim
	33	57	M	u	t12	2.77	1.45	9	17	18	145	126	2.5	2.7	no	sim
	34	53	M	u	11q	3.89	2.8	5	64	73	102	94	2.4	2.8	fcr	sim
	35	68	F	m	t12	3.04	2.23	8	76	101	244	132	2.9	3.2	BKi	sim
	36	71	F	u	17p	3.25	2.04	2	5	4	170	136	3.6	2.7	BKi	sim
	37	69	F	m	13q	4.41	2.89	4	72	74	390	248	2.5	2.5	no	sim
	38	57	M	m	na	4.48	2.01	2	4	4	142	126	1.7	1.8	no	ros
	39	64	F	m	13q	4.21	2.5	10	50	53	238	233	1.8	2.1	no	sim
	40	67	F	m	na	3.6	1.94	18	80	116	226	242	3.1	3.6	no	sim
	41	63	F	u	na	4.49	2.23	12	148	184	162	144	3.7	5.4	BKi	sim
	42	41	M	u	11q	4.78	2.83	7	59	52	90	78	3.6	4.8	BKi	sim
	43	76	F	m	na	4.1	2.05	7	43	46	230	222	1.8	1.8	no	ros
	44	67	M	m	13q	3.1	2.14	5	161	152	76	73	2.6	2.8	no	sim
	45	68	F	u	17p	5.52	3.3	3	167	174	231	207	3.7	3.7	BKi	sim
	46	38	M	na	na	2.93	1.44	3	47	42	149	132	1.5	1.4	fcr	sim
	47	84	F	na	13q	3.45	1.94	2	92	64	110	127	3.5	3.8	CO	sim
	48	76	M	u	neg	3	1.99	2	77	76	120	103	4.8	5.4	BKi	sim
	49	55	F	m	13q	4.04	1.86	3	280	315	132	142	2.1	2.4	fcr	sim
	50	65	M	m	na	3.56	2.36	12	19	21	146	144	1.5	1.5	no	sim
	51	48	F	u	t12	3.2	2.35	3.5	18	31	173	145	2.9	3.2	BKi	sim
	52	63	M	u	neg	3.13	1.92	3	28	30	142	137	2	2.5	fcr	sim
	53	75	F	m	na	2.6	1.83	5	15	17	237	214	2.5	2.5	no	sim
	54	72	M	m	t12	2.4	2.16	6	175	247	187	170	5.6	7.2	Ven	sim
	55	70	M	u	11q	3.03	1.96	3	18	13	276	131	2.3	2.5	BKi	ros
	56	66	F	m	13q	4.2	1.88	5	54	62	175	149	3.2	3.6	BKi	sim
	57	69	F	u	neg	2.67	1.77	6.5	140	194	180	155	4	3.8	BKi	sim
Avg		64.2				3.6	2.1	6.4	68.6	80.2	177.3	154	2.8	3.0		
SE		1.1				0.1	0.05	0.6	9.0	9.7	9.6	8.1	0.1	0.2		
**Progressor no statins**																
	P1	64	F	u	13a	2.5		68	18	28	175	190	1.8	1.8	BKi	
	P2	82	M	u	neg	1.9		38	20	183	136	117	2.4	5.2	BKi	
	P3	76	M	m	13q	1.4		89	69	41	115	84	2.5	5.3	CO	
	P4	58	F	u	t12	2		36	9.2	48.5	104	78	1.9	4.1	BKi	
	P5	75	M	m	13q	2.63		38	48	93	173	162	1.5	2.5	BKi	
	P6	74	M	u	neg	3.1		51	16	37	152	111	1.8	5	FR	
	P7	60	M	u	neg	3.65		23	12.3	48	117	77	2.4	5.2	BKi	
	P8	59	F	u	neg	3.8		32	17	125	229	236	1.7	3.9	BKi	
	P9	78	M	na	t12	2.1		39	49	78	56	52	3.9	6.5	CO	
	P10	76	M	u	17p	2.3		10	19	140	134	99	4.4	10.1	BKi	
	P11	73	F	u	13q	1.9		18	38	142	150	65	2.7	5.8	BKi	
	P12	58	M	u	11q	1.7		14	29.4	219	140	86	3.1	6.2	BKi	
	P13	56	F	m	13q	2.76		27	325	325	235	160	2.8	3.5	VO	
	P14	69	F	na	na	1.6		161	22	12.1	167	54	2.4	7.6	BKi	
	P15	78	M	u	17p	4		15	17	81	203	198	3.7	5.4	BKi	
	P16	65	F	u	11q	3.3		31	18.7	114	180	133	2	3.5	BKi	
	P17	56	M	u	na	4.76		52	25	329	195	143	1.8	2.6	BKi	
	P18	84	F	m	13q	2.95		82	60	120	219	167	2.6	4.6	no	
	P19	62	F	u	na	3.6		25	96	228	308	156	2.8	4.7	no	
Avg		68.6				2.7	*	44.7	47.8	125.9	167.8	124.6	2.5	4.9		
SE		2.1				0.2		8.2	16.3	21.6	13.0	12.1	0.2	0.4		
**Benign no statins**																
B	1	61	M	m	na	2.91	2.99	81	32	27	166	165	1.8	2.3	no	
B	2	62	M	m	na	1.63	2.74	89	9	13	204	191	1.8	2.3	no	
B	2	55	F	m	na	1.78	2.6	82	12	15	163	132	1.8	1.7	no	
B	4	59	F	m	na	1.67	1.69	112	40	65	187	180	1.6	2	no	
B	5	64	F	m	na	2.2	1.98	63	18	17	306	260	2.1	2.4	no	
B	6	84	F	m	na	3.68	3.93	117	50	99	168	120	3.7	4.5	no	
B	7	71	M	m	13q	2.37	2.95	20	100	111	173	191	2.1	2	no	
B	8	52	M	m	na	2.82	2.09	17	9.4	10.6	140	153	2.1	2.5	no	
B	9	76	F	m	na	3.5	3.08	37	10	12	175	146	2.4	3	no	
B	10	63	M	m	13q	3.3	2.3	98	46	35	203	155	2.5	2.9	no	
B	11	85	F	m	na	3.2	3	66	28	31	185	161	2.3	2.7	no	
B	12	56	F	m	na	2.66	2.91	67	8	13	214	300	1.5	1.7	no	
B	13	72	F	m	na	3.93	4.08	119	17	31	204	208	2.2	2.4	no	
B	14	54	F	m	na	2.87	2.92	84	28	25	252	307	1.7	2	no	
B	15	52	F	m	na	2.02	1.96	115	12	15	236	262	1.3	1.6	no	
B	16	61	M	m	na	2.21	2.25	122	11	21	185	126	1.8	2.1	no	
B	17	73	F	m	na	2.63	3.65	90	17	20	245	229	2.4	2.3	no	
B	18	73	F	m	na	3.63	2.86	155	10	19	219	204	1.9	2.4	no	
B	19	78	M	m	na	3.1	3.76	77	11	25	275	187	2.4	3	no	
B	20	78	F	na	na	2.73	2.9	66	17	18	178	175	1.7	2.1	no	
B	21	59	F	m	13q	2.05	2.36	206	71	68	96	76	2.3	2.7	no	
Avg		66.1				2.7	2.8	89.7	26.5	32.9	198.8	187.0	2.1	2.4		
SE		2.3				0.2	0.1	9.4	5.2	6.2	10.2	12.8	0.1	0.1		

*Note:* *The final LDL reading for the progressor group is not recorded as it was often not available around the time of instituting first‐line therapy.

Abbreviations: Age = age of patient in years at time of statin institution, BKi = BTK inhibitor including acalabrutinib, ibrutinib, zanubrutinib, C = chlorambucil, FCR = fludarabine, cyclophosphamide, rituxan, FISH = fluorescence in situ hybridization: na = not available; neg = negative; t12 = trisomy 12, FR = fludarabine, rituxan, *IGHV*: m = mutated; u = unmutated, LDL = low‐density lipoproteins (mM), No = remains untreated, Obinutuzumab CO = chlorambucil, Platelets = ×10^9^ cells/L, Pre = prior to institution of statin; Post = at time of analysis of response to statins, Rituxan BR = bendamustine, Ros = rosuvustatin, Sex: M = male; F = female, Sim = simvastatin, Time = months between pre‐LDL and post‐LDL measurement in months, Tx = ultimate first‐line treatment, Ven = venetoclax, WBC = white blood cell count (mainly lymphocytes) × 10^9^ cells/L, β2M = beta‐2‐microglobulin (μg/mL).

**FIGURE 1:**
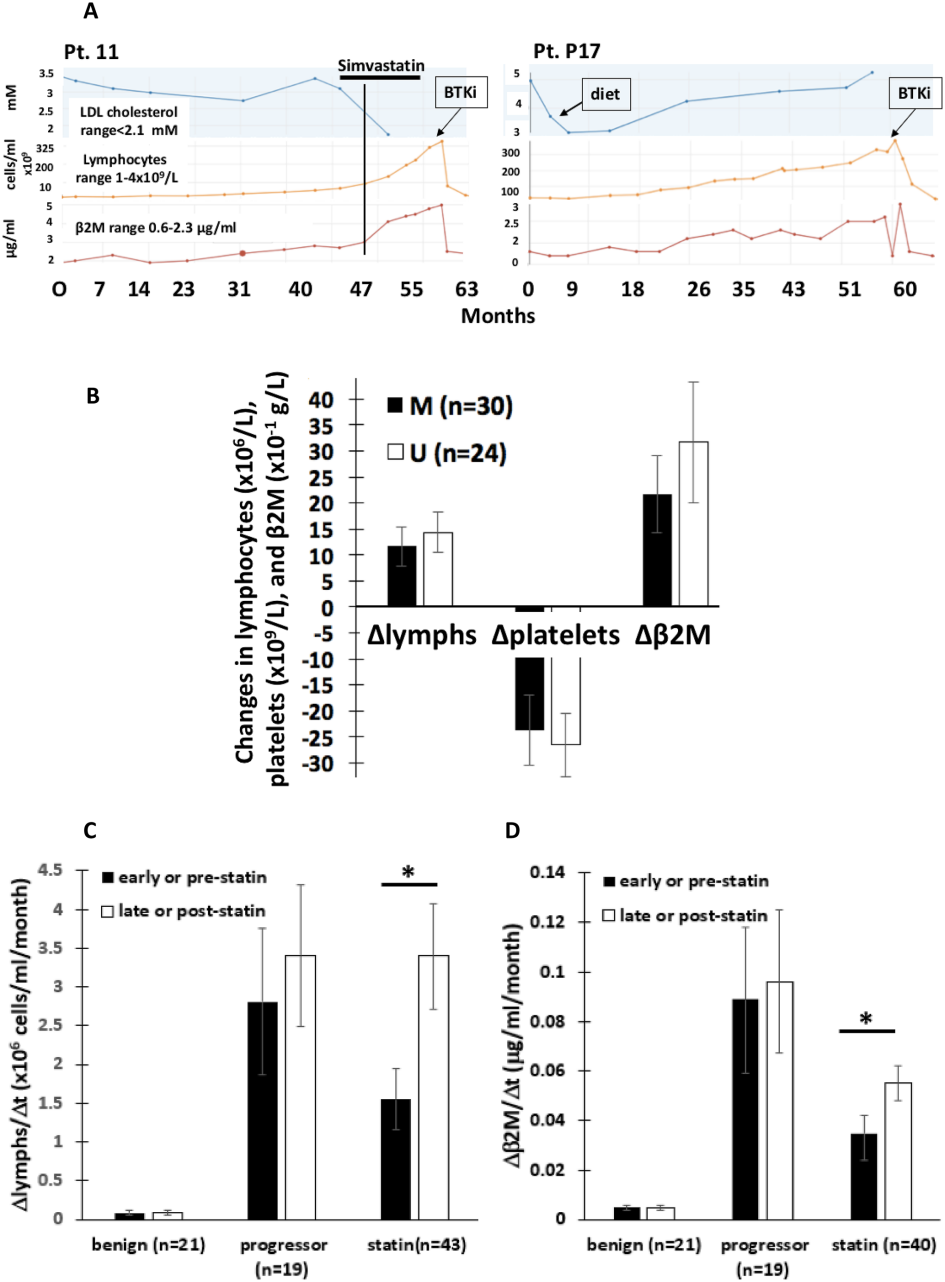
Markers of CLL progression after statin institution. Clinical records of 57 patients (26 female and 31 male) who commenced statins between 2012 and 2023 in the “watch and wait” phase of management with documented serial LDL measurements, were examined. Records of 19 patients with progressive disease (eventually requiring first‐line therapy) and 21 patients with benign disease (with no evidence of disease progression after prolonged follow‐up) that also had multiple recorded LDL measurements were used as controls. (A) Temporal changes in lymphocytes, β2M, and LDL cholesterol are shown for Patient 11 treated with simvastatin (left panel) and patient P17, who progressed to first‐line therapy but was never exposed to statins (right panel). Note the inflection point indicated by the vertical line seen shortly after starting simvastatin. (B) Lymphocytes, platelets, and β2M values were recorded prior to starting the statin and after the LDL cholesterol had dropped near 2.1 mM. Averages and standard errors of the differences are shown for patients with U‐CLL and patients with M‐CLL. *IGHV* mutation status was not available for three patients. (C, D) Rates of change of lymphocyte numbers (C) and β2m levels (D) before and after statins were calculated from the difference in these parameters divided by the time in months between the two measurements for 43 and 40 patients, respectively, where these rates could be reliably calculated. Rates for benign and progressor patients not treated with statins were calculated before and after a spontaneous inflection point. Otherwise, “early” and “late” results were the rate changes observed over the entire observation period. The results are consistent with mild progression of CLL following institution of statins. *, *p* < 0.05. statistical analysis was done by Student *t*‐tests or ANOVA.

Clinical records of another 40 patients with recorded LDL measurements but never treated with statins were available to be used as a control group. This group might help address the possibility that inflections in lymphocyte and β2M rates observed with statins (Figure 1A) were simply epiphenomena associated with CLL evolution. The patients were classed into two groups on the basis of the outcome at the end of the observation period. Twenty‐one had “benign” disease [10] and did not develop symptoms or require treatment over many years. Nineteen had progressive disease and developed indications for therapy in the observation period [1, 10]. These benign and progressor patients are described in Table 1.

Baseline LDL levels for these patients were lower than for statin‐treated patients (Table 1). As expected, lymphocytes and β2M levels increased while platelets decreased at the end of the observation period in the progressor patients but did not change significantly in the benign group. Inflections in lymphocyte and β2M slopes were not seen in benign patients and in only three of 19 progressor patients. In contrast, such inflections were observed in over 50% of statin‐treated patients (Figure 1A, left panel). The course of most progressor patients is exemplified by patient P17 (Figure 1A, right panel) with a gradual sustained rise in lymphocyte counts and β2M levels. Interestingly, this patient transiently lowered his LDL levels from 5.3 to 3.1 mM by life‐style changes without obviously changing lymphocyte and β2M slopes (Figure 1A, right panel). The rates of change of lymphocyte counts and β2M levels were calculated before (“early”) and after (“late”) these inflection points. Otherwise, the slopes over the entire observation period were used to represent “early” and “late” rates. These rates were significantly lower in the benign patients and did not change significantly in progressor patients (Figure 1C,D).

A provocative preliminary suggestion from these observations is that statins as single agents may induce mild progression of CLL. How statins might cause CLL cells to become more aggressive is unclear. Institution of simvastatin was previously shown to be associated with decreased phosphorylated STAT3 proteins in CLL cells in vivo [11]. This phenotype has since been linked to more aggressive clinical behavior [12]. A tumor‐promoting effect of statins may not have been noted previously because it is mild and would likely have been attributed to natural disease progression. Moreover, it may be masked by low blood concentrations of statins with conventional dosing regimens that do not change LDL concentrations significantly. The effect may also have been overcome by downstream mevalonate pathway components supplied in the diets of some patients [3]. Regardless, the findings reported here suggest that previous reports linking statin use with prolonged PFS in CLL [5, 6] may reflect lead time bias or other problems associated with epidemiologic studies involving low numbers of subjects [9]. Instead, statins as single agents do not apparently slow progression of CLL and may even modestly stimulate its growth.

The small sample size of the cohort and its retrospective and descriptive nature are limitations of this study. Most patients were treated with simvastatin, and it is not clear whether the results apply to other statins. Other drawbacks are the lack of uniform time points to assess clinical responses as well as correlative mechanistic studies to explain the observations. However, the study population is uniquely informative in that sequential lipid profiling was available to ensure the statin had biological activity. Larger studies are needed to determine whether statins, as single agents, are inactive or could possibly pose some harm to patients with CLL. Preclinical studies and interventional trials are needed to realize the potential therapeutic benefits of statins in CLL.

## Author Contributions

D.E.S. conceived the project and wrote the manuscript.

## Conflicts of Interest

The author declares no conflicts of interest.

## Data Availability

The data that support the findings of this study are available on request from the corresponding author. The data are not publicly available due to privacy or ethical restrictions.
